# The middle fossa approach with self-drilling screws: a novel technique for BONEBRIDGE implantation

**DOI:** 10.1186/s40463-019-0354-7

**Published:** 2019-07-29

**Authors:** Peng You, Lauren H. Siegel, Zahra Kassam, Matthew Hebb, Lorne Parnes, Hanif M. Ladak, Sumit Kishore Agrawal

**Affiliations:** 1 Department of Otolaryngology-Head and Neck Surgery, Schulich School of Medicine & Dentistry, Western University, London, Canada; 2 Department of Medical Imaging, Schulich School of Medicine & Dentistry, Western University, London, Canada; 3 Department of Clinical Neurological Sciences, Schulich School of Medicine & Dentistry, Western University, London, Canada; 4 Department of Medical Biophysics, Schulich School of Medicine & Dentistry, Western University, London, Canada; 5 University Hospital, Room B1-333, London Health Sciences Centre – University Hospital, 339 Windermere Road, London, Ontario N6A 5A5 Canada; 6 Department of Electrical & Computer Engineering, Faculty of Engineering, Western University, London, Canada

**Keywords:** Bone conduction implant, BONEBRIDGE, Middle fossa approach, Conductive hearing loss, Surgical technique, Implants

## Abstract

**Background:**

Bone conduction implants can be used in the treatment of conductive or mixed hearing loss. The BONEBRIDGE bone conduction implant (BB-BCI) is an active, transcutaneous device. BB-BCI implantation can be performed through either the transmastoid or retrosigmoid approach with their respective limitations. Here, we present a third, novel approach for BB-BCI implantation.

**Objective:**

Describe the detailed surgical technique of BB-BCI implantation through a middle fossa approach with self-drilling screws and present preliminary audiometric outcome data following this approach.

**Methods:**

A single institution, retrospective chart review was completed for patients implanted with the BB-BCI via the middle fossa approach. Preoperative planning and modelling were performed using 3D Slicer. Audiological testing was performed pre- and post-operatively following standard audiometric techniques.

**Results:**

Forty patients underwent BB-BCI implantation using the middle fossa approach. Modelling techniques allowed for implantation through the use of external landmarks, obviating the need for intraoperative image guidance. The surgical technique was refined over time through experience and adaptation. Mean follow-up was 29 months (range 3–71 months) with no surgical complications, favourable cosmesis, and expected audiometric outcomes. An average functional gain of 39.6 dB (± 14.7 SD) was found.

**Conclusion:**

The middle fossa technique with self-drilling screws is a safe and effective option for BONEBRIDGE implantation. As a reference for other groups considering this approach, an annotated video has been included as a supplement to the study.

**Electronic supplementary material:**

The online version of this article (10.1186/s40463-019-0354-7) contains supplementary material, which is available to authorized users.

## Introduction

Bone conduction implants (BCI) rely on vibratory excitation of the temporal bone which in turn stimulates the cochlea. These implants are used when conventional hearing aids cannot be worn because of medical or anatomic conditions such as recurrent otitis externa, aural atresia, and single-sided deafness (SSD). In the case of SSD, the sound is transmitted to the better hearing ear via bone conduction [1–3].

BCIs can be broadly categorized as percutaneous, passive transcutaneous, or active transcutaneous. The bone anchored hearing aid (BAHA) is a commonly used percutaneous BCI [3]. The BAHA stimulates the temporal bone through a surgically implanted, osseointegrated titanium screw that is attached to an external sound processor. While BAHAs show favourable audiological outcomes, the disadvantages of the skin-penetrating implant include possible infection, wound dehiscence, fixture losses, and/or need for revision surgery [4]. The BAHA complication rate is higher for pediatric patients than adults [4]. Furthermore, up to 11% of BAHA candidates refuse implantation over concerns of aesthetic and social acceptance surrounding the percutaneous abutment [5].

In comparison, transcutaneous BCIs differ in that the skin overlying the implanted device is intact. For passive transcutaneous BCIs such as the BAHA Attract (Cochlear Ltd., Sydney, Australia) and Sophono (Sophono Inc., Boulder, CO, USA) [6], both the actuator and audio processor are located within an external housing. The external vibration is then transmitted transcutaneously to an osseointegrated implant that is covered by skin. These devices avoid a percutaneous abutment and associated complications. However, they require significant contact forces and generate less gain than the percutaneous devices [7]. The force exerted by the sound processor may also cause pressure marks or skin pain, which has been associated with reduced device adherence [8].

**Fig. 1:**
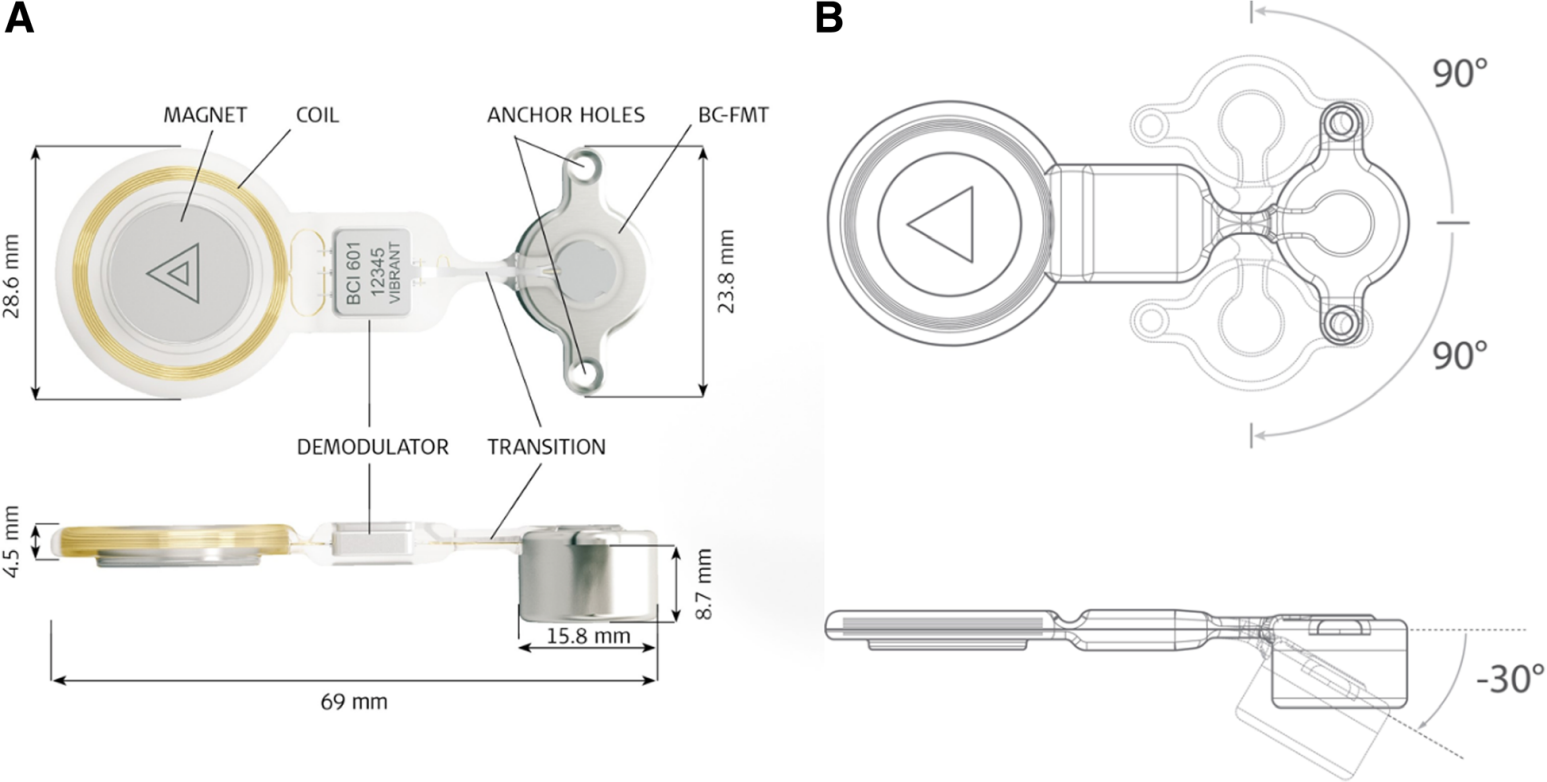
**a** BONEBRIDGE bone conduction implant with dimensions in top and side view. **b** Flexible transition segment of BONEBRIDGE may be bent +/− 90 degrees in the horizontal plane and − 30 degrees in the vertical plane. Image courtesy of MED-EL GmBH

Active transcutaneous BCIs, such as the BONEBRIDGE bone conduction implant (BB-BCI), are semi-implantable so that the vibratory energy does not need to be transmitted through the skin [6]. The BB-BCI system consists of two components: an internal implant housing the Bone Conduction-Floating Mass Transducer (BC-FMT) and the external audio processor (Fig. 1). Since the BC-FMT generates the vibratory energy, the small external audio processor only houses the microphone and batteries, similar to cochlear implants. This avoids the setbacks of the BAHA’s percutaneous abutment, as well as the skin pressure and vibration attenuation seen with passive transcutaneous BCIs. However, these implants can be surgically challenging to implant due to the size of the BC-FMT.

The BB-BCI first launched in Europe in 2012 [9], and the first North American implantation took place in April 2013 at the London Health Sciences Centre, London, Ontario, Canada [10].

Health Canada has approved this system for patients over the age of 5 years with conductive or mixed hearing loss and bone conduction thresholds of ≤45 dB HL (decibels hearing level) at 0.5-3 kHz. The BB-BCI is also an option for patients with SSD, where the contralateral ear has a hearing threshold between 0 and 20 dB HL measured at 0.5-3 kHz [11]. In July 2018, the BB-BCI was granted “de Novo clearance” from the United States (US) Food and Drug Administration and the indications may continue to evolve. As it stands, the audiologic criteria are the same as above, but the US indication for implantation is currently for patients 12 years or older. Moreover, bilateral fitting of the BB-BCI can be considered for patients with a symmetrically conductive or mixed hearing loss. The interaural difference in bone conduction thresholds should be less than 10 dB on average at 0.5-3 kHz or less than 15 dB at individual frequencies [12].

The commonly used approaches to BB-BCI implantation are the transmastoid or retrosigmoid techniques, where the BC-FMT is positioned in the sinodural angle or behind the sigmoid sinus, respectively [13]. A disadvantage of the BB-BCI lies in the sizeable physical profile of its BC-FMT (15.8 mm × 8.7 mm), and sufficient space must be found in the temporal bone to house the implant. While more common, the transmastoid approach may not be feasible in sclerotic anatomy, mastoid cavities, or individuals with chronic otitis media [14]. In comparison, the retrosigmoid approach must take into account the curvature of the skull and dissection of nuchal musculature [11]. A second notable disadvantage with the BB-BCI is that the drill bit supplied by the manufacturer uses a dental coupling. Unfortunately, this drill bit is not compatible with most North American drill systems, so the self-tapping screws included in the implant set cannot be used. For these reasons, a novel middle fossa approach was developed at our centre [11, 15] and refined over the first 40 cases. This paper provides a detailed description of the middle fossa approach with self-drilling screws for BB-BCI implantation. Preoperative modelling will be highlighted, including a method to obviate the need for image guidance for intraoperative landmarks. Finally, the surgical outcomes of patients implanted with this technique will be presented.

## Materials and methods

### Surgical device

The BB-BCI consists of an implantable transducer and an external audio processor (Amadé or Samba). The implanted portion is intended to lie entirely underneath the skin and attach to the processor with a magnet. The implantable section consists of a receiver coil, the demodulator, and the BC-FMT. The BC-FMT represents the most substantial aspect of the device and sits in a casing that is 15.8 mm in diameter and 8.7 mm in depth. The device is anchored to the temporal bone with screws located 23.8 mm apart (Fig. 1a). Of note, a flexible transition segment separates the coil and transducer section. Therefore, the device can be adjusted 90 degrees to either side for a custom configuration. The BC-FMT can also be pivoted 30 degrees medially to adapt to the curvature of the skull (Fig. 1b).

### Retrospective chart review

A single institution, retrospective chart review was completed of all patients implanted with the BB-BCI via the middle fossa approach. Patient charts were reviewed to summarize OR (operating room) notes, determine follow-up dates, document the incidence of adverse events following the procedure, and collect pre- and post-operative audiometric data. Unaided air conduction and bone conduction thresholds were measured pre-operatively. Aided thresholds were measured in the sound field with the BB-BCI in use and appropriate masking was used for the unimplanted ear. This study was exempted from approval by the institutional review board because all data were collected for quality assurance purposes and kept anonymously.

### Preoperative planning and modelling

For modelling, the free and open source 3D Slicer software was used to plan the BB-BCI placement (https://download.slicer.org/) [16]. The software runs on modern Windows, Mac OS X (10.7 and up), and a variety of Linux platforms.

Preoperative computed tomography (CT) scanning allowed for the assessment of temporal bone thickness. In the middle fossa approach, imaging helps ensure the squamous temporal bone thickness is adequate to house the BC-FMT. In general, the lowest part of the squamous temporal bone thickens near the middle fossa floor, marking the ideal location to house the BB-BCI. This was initially verified by scanning a set of cadaveric temporal bones and performing implants in the temporal bone laboratory.

The modelling begins by loading the DICOM data of thin-sliced dedicated temporal bone scans into the 3D slicer. The desired image series corresponding to the side of implantation is loaded into the software. If a localizer image is present, which usually appears as the first image of the series, it must first be deleted in the raw DICOM directory before importing the DICOM folder.

To display the three-dimensional (3D) temporal bone model, the *Conventional* layout under the view setting is often the easiest to work with. Next, the *Volume Rendering* module under the module drop-down menu is selected to begin creating the 3D model. Looking at the module interface located on the left side of the screen, the layer visibility (eye) icon next to the current volume can allow the 3D temporal bone model to be displayed. To improve processing time, rendering can be changed from *VTK CPU Ray Casting* to *VTK GPU Ray Casting*. Finally, under *Display*, the pre-set can be changed to *CT-AAA* for better colour rendering. The shift slider helps to toggle between soft tissue and bone views. With the 3D model constructed, one can rotate it in space to view critical structures, such as the sigmoid sinus. Other options include displaying the *Region Of Interest (ROI)* layer under *Display* and cropping the model in any of the three axes.

**Fig. 2:**
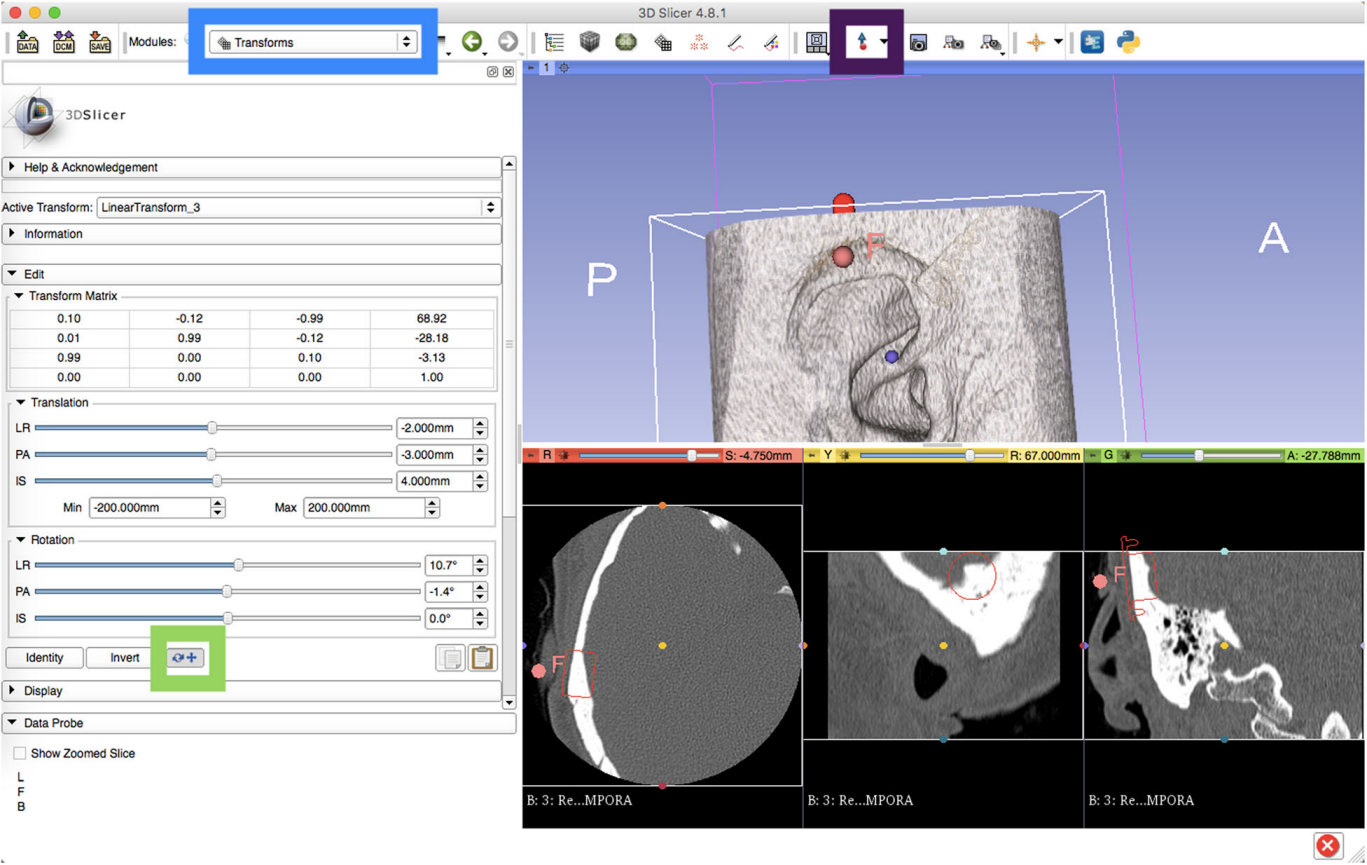
Screen capture of the 3D Slicer interface in Conventional view for preoperative planning of middle fossa approach to BONEBRIDGE implantation. Traditional axial, sagittal, and coronal views of a temporal bone CT are visible at the bottom of the screen. The BC-FMT is highlighted with a red outline on these slices, and a red fiducial marks the centre of the implant on the skin. Blue box = module drop down menu. Green box = option to select Transform selection to alternate between global versus regional transform. Purple box = option to add fiducials. F = digital fiducial

Planning the positioning of the BB-BCI involves overlaying the 3D model of the implant. The model template is available from MED-EL GmBH (BC-FMT_3D_Template.vtk) upon request. The template file is then added into the scene through the software’s *Add Data* option. Further editing of the implant model is done by selecting *Models* in the module drop-down. We favour changing the display colour to red to make the model easily visible. Furthermore, we enable *Slice Display* to visualize the model outline within the traditional CT images (Fig. 2).

Positioning the template module is performed through the *Transforms* module. After creating a *New Linear Transform* within the *Active Transform* menu, the BC-FMT_3D_Template file is moved from the *Transformable* to the *Transformed* list under the *Apply Transform* menu. The BC-FMT 3D model is now able to be positioned in various planes. For ease of use, we found translation in the local reference frame to be more intuitive. The option to change between translation in global versus local reference frame can be found near the bottom of the *Edit* drop-down menu (Fig. 2). Next, the model is positioned at the thickest possible portion of the middle fossa bone using the various *Translation* and *Rotation* sliders. Scrolling through views on the axial or coronal CT slices can be used for confirming placement. The temporal bone can be rotated to view the medial surface, where the groove of the transverse and sigmoid sinus are readily visible.

**Fig. 3:**
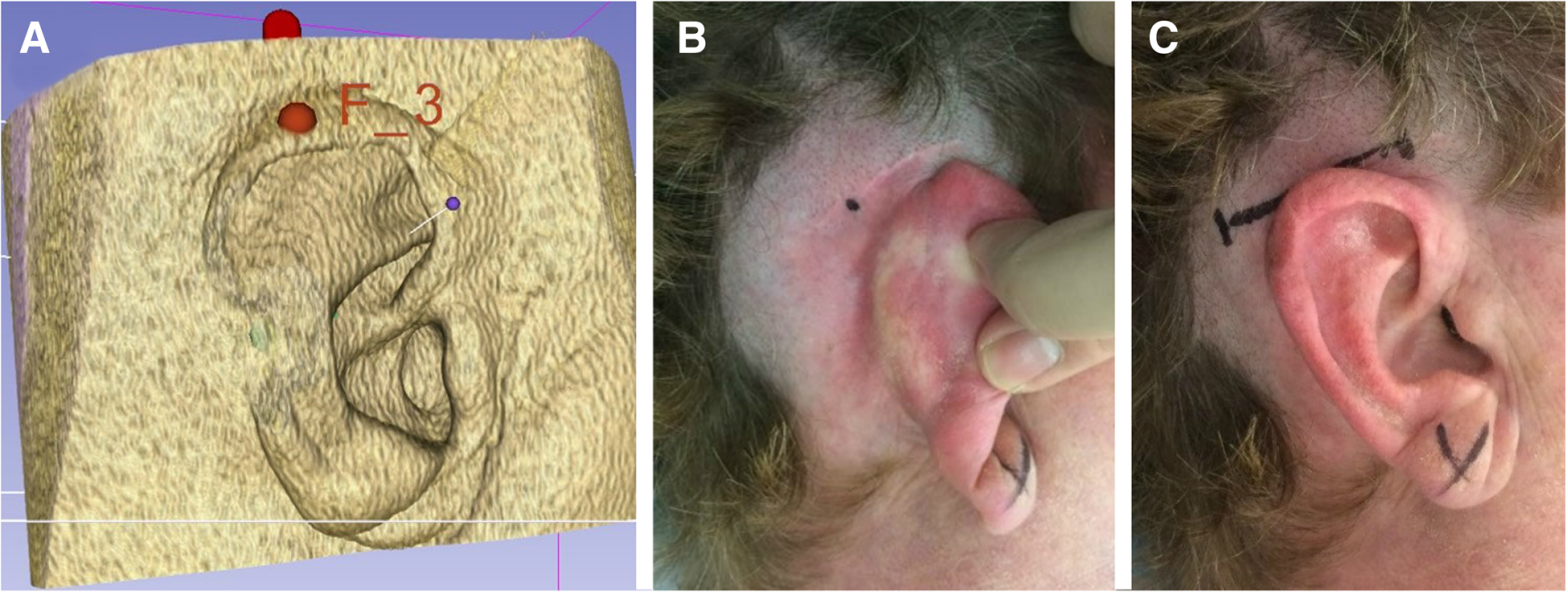
Middle fossa approach to BONEBRIDGE implantation following preoperative planning. **a** Final digital placement of implantation with the corresponding fiducial maker. **b** The 3D model marker is referenced to the auricle and a skin marker is made. **c** Horizontal incision designed across skin marker

Finally, with the BC-FMT model in its appropriate position, we aim to recreate the external landmark to be used during surgery. The 3D slicer software allows the user to place a digital fiducial (Fig. 2). After displaying the center of the implant on the axial view, a digital fiducial is placed at the overlying soft tissue. After this, the user returns to *Volume Rendering* and toggles back to the soft tissue window. The modelling process thus allows the creation of a reference point relative to the external ear. By using external landmarks, intraoperative image guidance is not required. The final image (Fig. 3a) is then printed and brought to the operating room. The centre of the implant is referenced according to the position of the auricle, and then the point is labelled on the patient using a skin marker.

## Results

### Surgical technique

A horizontal 3 cm incision is marked above the ear and centred on the implant position determined using 3D slicer (Fig. 3c). The incision is placed in the hairline to improve post-operative cosmesis. The temporalis muscle is divided horizontally. Using a periosteal elevator, the pocket to house the receiver coil is developed based on the final position of the implant. This is verified using the sizing template included in the BCI sizer kit. Once the temporal bone is encountered, a cylindrical craniotomy is drilled to easily accommodate the BC-FMT. Of note, the BC-FMT itself does not need to contact bone as all the vibrational energy is transmitted through the two anchoring screws. Therefore, the craniotomy should be sized slightly larger than 16 mm to allow for free movement of the BC-FMT, while preserving enough bone to place the anchoring screws. The added movement will enable a slight tilting of the BC-FMT and wings to accommodate for the curvature of the skull.

**Fig. 4:**
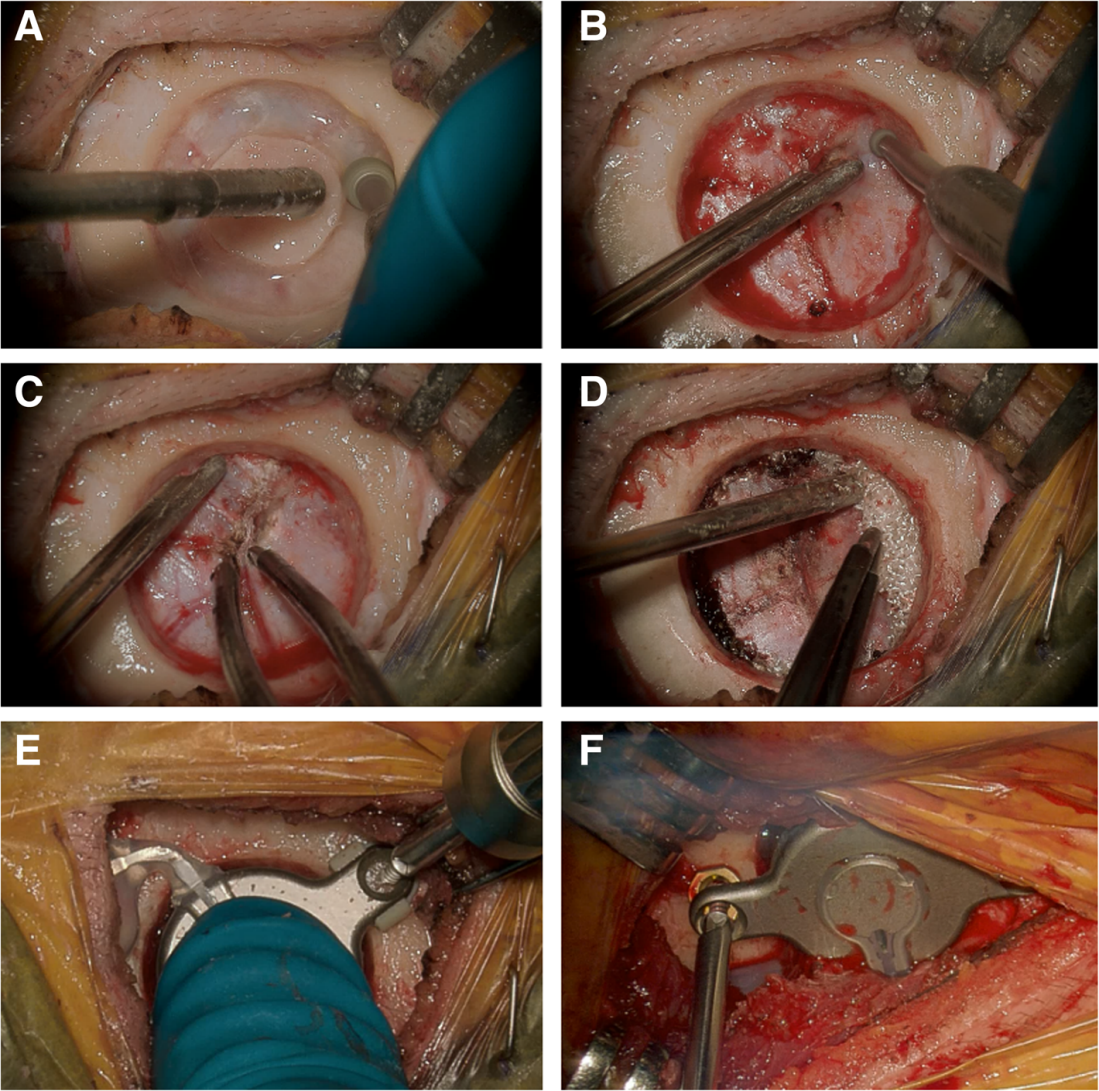
Intraoperative pictures of middle fossa approach to BONEBRIDGE implantation with self-drilling screws. **a** Outline receiver well for implant using a standard otologic drill. **b** A smaller otologic drill is used to remove bone down to the dura to facilitate a vertical wall for the cylindrical craniotomy. **c** Small dural vessels are addressed with bipolar electrocautery. **d** Surgicel is used for hemostasis and slight tenting of dura away from the craniotomy. **e** Implant secured using self-tapping screws and BONEBRIDGE lifts. **f** Intraoperative picture from a patient before the availability of dedicated lifts. Rings cut from Synthes MatrixMIDFACE plates were purposed as custom lifts

Two options are available to create the cylindrical craniotomy. First, a neurosurgical trocar (14 mm) can be used followed by standard otology bone drills to create the appropriate recess [17]. This was performed initially at our centre, but it was found that in experienced hands, using otologic burrs for the entire opening could be accomplished in a similar time (Fig. 4a). Irrespective of the drilling technique, bone is removed down to the dura to facilitate placement and adjustment of the implant (Fig. 4b). The dura is then freed from the bone edges, and small rolls of Surgicel Absorbable Hemostat (Johnson and Johnson, Slough, UK) are placed circumferentially to help tent the dura away from the craniotomy and provide hemostasis. Small dural vessels, if encountered, can be easily controlled with bipolar cautery and bone wax. Typically, a smaller diamond burr is used to ensure the edges of the craniotomy are completely vertical (perpendicular to the dura). The transducer sizer provided with the surgical kit should be used to ensure an adequate fit.

An annotated video has been included as a supplement to the study (Additional file 1). The video summarizes the various surgical steps for the middle fossa approach, including the device positioning and self-drilling screws subsequently detailed.

### Device positioning

**Fig. 5:**
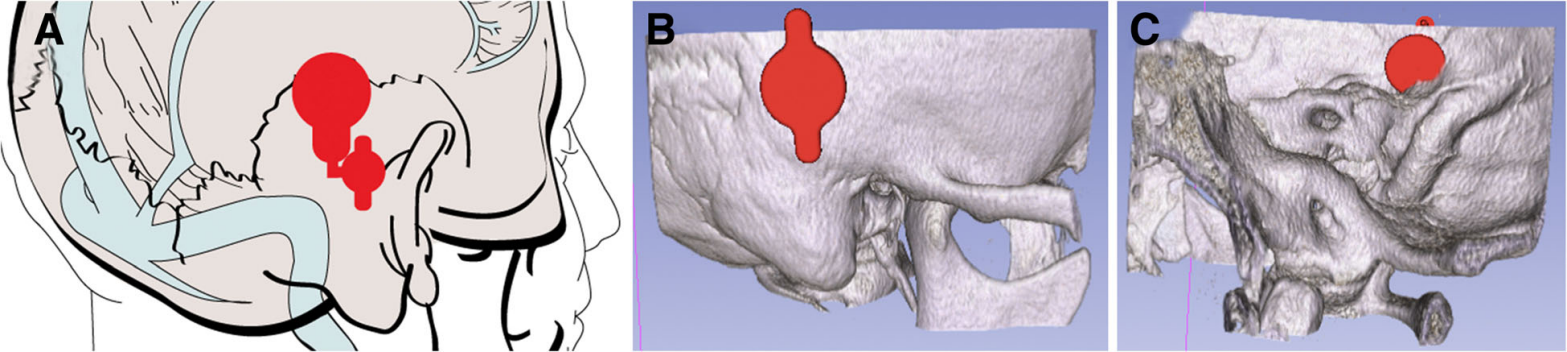
**a** Placement of BONEBRIDGE in the middle fossa anterior to the transverse-sigmoid junction. The anchor screws are oriented vertically with the transition section bent at 90 degrees to reduce the vertical profile of the device and to bring it closer to the auricle. Lateral (**b**) and medial (**c**) view of the 3D slicer model with the implant and the temporal bone

**Fig. 6:**
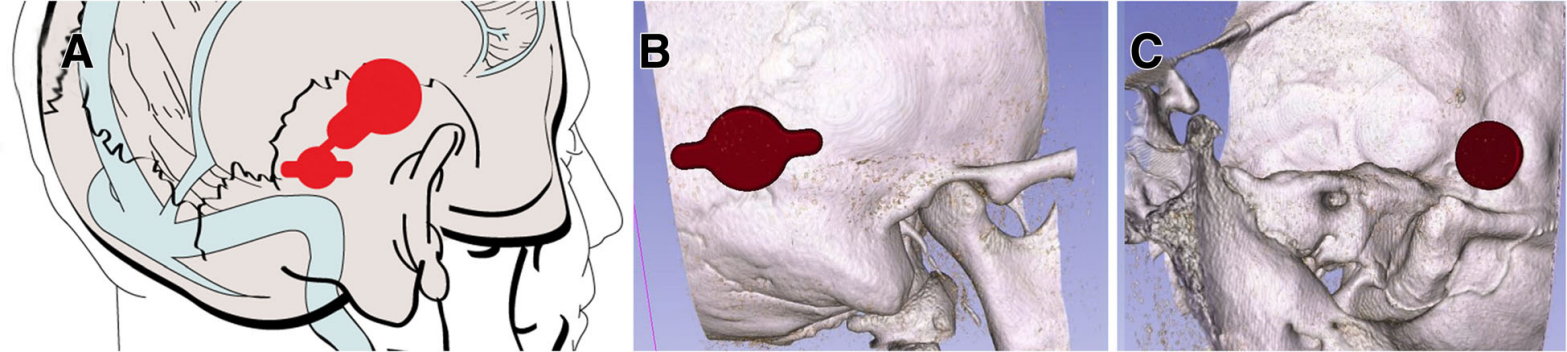
**a** Placement of BONEBRIDGE in the middle fossa superior to the sigmoid sinus. The transition section is bent at 45 degrees to bring the coil assembly closer to the auricle. Lateral (**b**) and medial (**c**) view of the 3D slicer model with the implant and the temporal bone

The thickness of the bone determines device positioning in the middle fossa. The thickest portion of the squamous temporal bone typically occurs either just anterior to the transverse-sigmoid junction (Fig. 5), or further posteriorly just superior to the transverse sinus (Fig. 6). For devices placed anterior to the transverse-sigmoid junction, the BB-BCI is typically placed with the screws in the vertical orientation. The device is then bent to 90 degrees at the transition segment to decrease the implant’s vertical profile and bring the coil assembly closer above the auricle. Conversely, for devices placed above the transverse sinus, the screws are oriented horizontally. The transition segment is then tilted 45 degrees anteriorly to bring the coil assembly closer to the ear (Fig. 6). The position of the coil assembly corresponds to that of the external audio processor, which when placed closer to the ear will bring the microphone closer to the natural hearing position.

### Lifts and self-drilling screws

The surgeon must balance between dural compression and lift from the skull when using the middle fossa approach. In cases where the bone thickness is less than 8.7 mm, the implant can be secured with spacers/lifts to avoid excessive compression to the dura. To address this, we initially used rings cut from the plates of Synthes MatrixMIDFACE Plating system (DePuy Synthes, West Chester, USA) to serve as spacers and to “lift” the BB-BCI (Fig. 4f). Since then, dedicated BCI Lifts have been developed by MED-EL and are available in 1, 2, 3, and 4 mm sizes [13].

To anchor the BB-BCI, the surgical implant kit includes 6 mm self-tapping cortical screws measuring 2 mm in diameter. Unfortunately, the accompanied drill bit with a dental coupling is not compatible with most North American drill systems, and therefore the self-tapping screws could not be used. Self-drilling screws (6 mm) from the Synthes MatrixMIDFACE Plating system were found to be appropriate substitutes.

When tightening the screws, the excessive torque requires attention. When a 6 mm screw is used, 3.9 mm of the tread is anchored to the bone while the remainder is within the profile of the implant’s wing. Initial experience with the self-drilling screws led to excess torque during the last millimetre of placement – this resulted in occasional fracture or stripping of the screw head. Therefore, a 1 mm BCI lift is always used with 6 mm self-drilling screws, allowing for 2.9 mm of tread within the bone, which is sufficient for stability and vibrational energy transfer. Similarly, a 3 mm BCI lift is used with 8 mm screws, although this combination has rarely been encountered in our experience. This technique also obviated the use of the included torque wrench. To ensure the best fit, the BC-FMT is held in place, and the anchoring screws are gently tightened in an alternating fashion. This allows the BC-FMT to be level and the screw heads to seat completely into the BCI wings.

### Surgical outcome

Forty consecutive patients underwent BB-BCI implantation using the middle fossa approach at our institution. There had been no complications since implantation with a mean follow-up of 29 months (range 3–71 months). Patients were discharged the day of the procedure, and implant activation occurred at approximately 2 weeks post-op.

### Audiometric outcome

Pure-tone averages (PTAs) were calculated as the average of the thresholds at frequencies 0.5, 1.0, 2.0 and 3.0 kHz for pre-operative air conduction and bone conduction (unaided conditions) and the post-operative aided condition (with BB-BCI in use). Functional gain was calculated as the difference between unaided air conduction thresholds and BB-BCI aided thresholds.

Mean air conduction PTA was 66.3 dB (± 14.6 standard deviation [SD]) and mean bone conduction PTA was 20.8 dB (± 14.0 SD). There was no change to residual hearing following BB-BCI implantation. The average functional gain overall was 39.6 dB (± 14.7 SD).

## Discussion

The BB-BCI can be offered to patients suffering from conductive or mixed hearing loss, as well as those with SSD, who cannot benefit from conventional hearing aids. Following the development at our centre, there are now three techniques for BB-BCI implantation: the transmastoid, retrosigmoid, and middle fossa approach [11, 14, 18].

The choice of the implantation approach is informed partly by a patient’s temporal bone anatomy and whether it will accommodate the size of the BC-FMT. The transmastoid approach is more frequently described in the literature [9, 11]. Typically, the retrosigmoid position is considered when there is insufficient mastoid space, previous mastoidectomy, or a history of chronic middle ear/mastoid infection that may jeopardize the implant. The challenges of the retrosigmoid approach include the more substantial curvature of the bone, separation of nuchal musculature during exposure, as well as the incision often extending below the hairline. Studies investigating post-operative pain following the retrosigmoid BB-BCI implantation are sparse. One study found the Headache Impact Test score to be higher in the retrosigmoid approach when compared to the transmastoid technique. However, the study was not powered for the subgroup comparison, and the results were not statistically significant [19]. Of note, a large body of literature suggests that the retrosigmoid approach in the context of vestibular schwannoma surgery is associated with more frequent postoperative headaches [20].

**Fig. 7:**
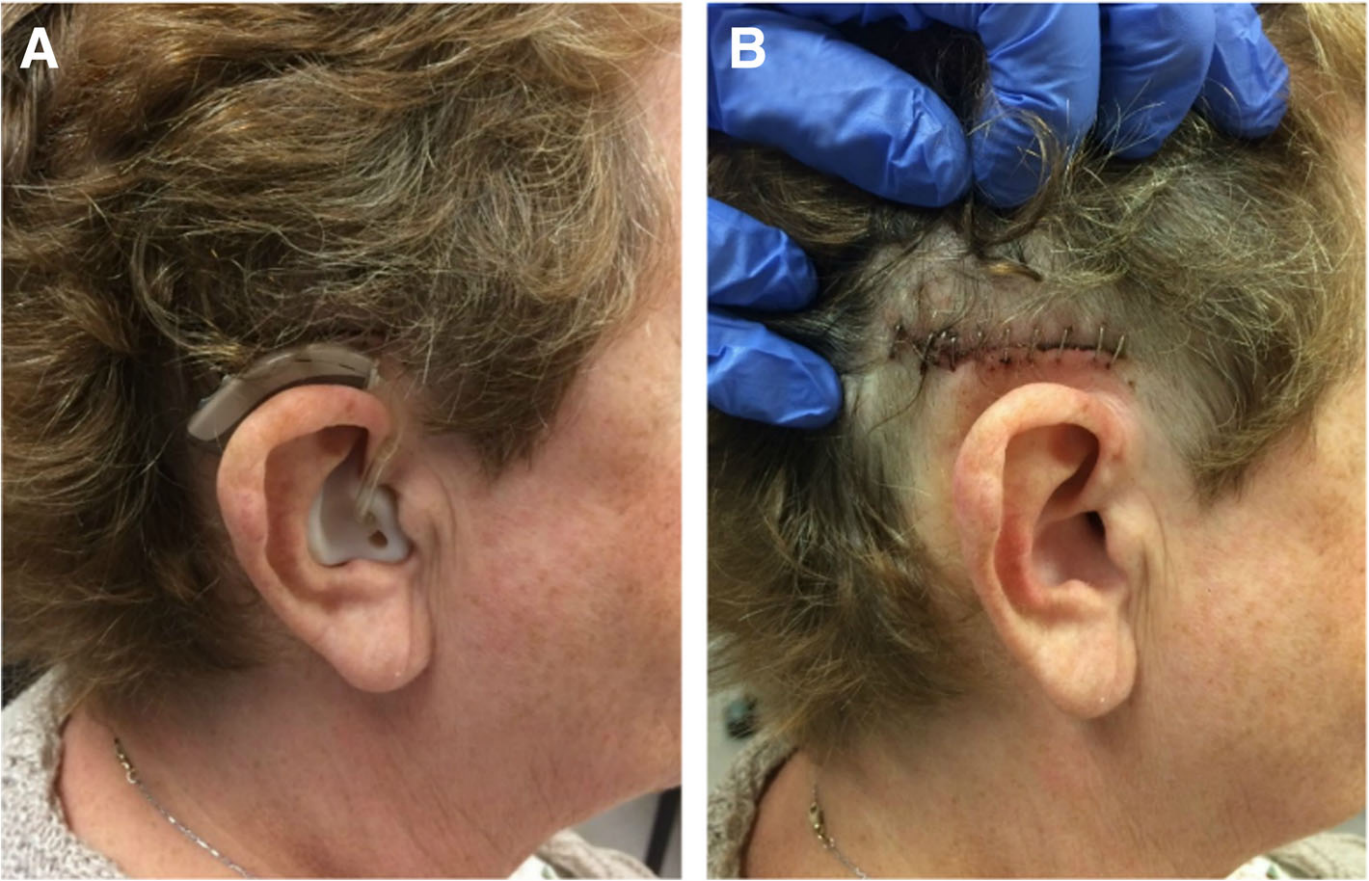
Results at post-operative day five following BONEBRIDGE implantation through the middle fossa approach. **a** A patient is wearing a hearing aid while awaiting activation. **b** The incision is camouflaged in the hairline with good cosmetic results

By comparison, the middle fossa technique involves a comparatively smaller incision. The incision is advantageous in its location within the patient’s hairline, optimizing scar cosmesis (Fig. 7). Moreover, the middle fossa approach avoids the disruption of air cells and nuchal musculature [11]. The novel middle fossa approach to BB-BCI implantation with self-drilling screws described in this paper has previously been presented and referenced [11], however, a formal technical paper had not yet been published.

In BB-BCI implantation, CT imaging is recommended as a standard preoperative assessment to ensure appropriate bony anatomy [9]. For instance, Law et al. examined pre-operative CT scans of 16 patients and found a high proportion of patients being considered for BB-BCI with contracted or operated mastoids, making them poor transmastoid candidates [21]. The authors also noted that three patients were radiographically unsuitable for both transmastoid and retrosigmoid approaches due to bone volume. At our centre, early patient evaluations for the middle fossa approach included magnetic resonance imaging (MRI)/CT fusion to map the details of the soft tissue and venous sinus. This is no longer performed because, from experience, CT alone was found to be sufficient in preoperative planning. Regarding the implant placement, other authors have proposed using image guidance or a custom template to assist in device positioning [22–24]. The unique modelling described presently uses digital external landmarks, which has obviated the need for any image guidance or custom templates. Experienced surgeons may opt not to perform any preoperative modelling if they are comfortable landmarking after a simple CT scan review.

BB-BCI implantation in general, and the middle fossa approach in particular, are limited by the thickness of the temporal bone. Unless larger BCI lifts are used, the height of the BC-FMT typically necessitates exposure of dura when the temporal bone is less than 8.7 mm. In our experience, using either a 1 mm or 3 mm lift provides a balance between dural compression and palpable height of the implant under the skin. Concerning the possibility that the BC-FMT may touch or press the meninges and cause discomfort, there has been no report to date that establishes a higher prevalence of headaches in the implanted population compared to the general population [11]. While no CSF leaks were encountered in our series, surgeons should be comfortable performing a dural repair if needed.

By extension, surgeons who wish to adopt the middle fossa approach must also be familiar with the temporal bone venous anatomy, specifically the transverse sinus. Bipolar cautery can be used to address other small dural veins encountered during the exposure. The surgeon should also be cautious of the vein of Labbé, which is a superficial anastomotic vein. This vein attaches medially and should not be affected by the minimal dural compression caused by the BC-FMT.

Self-tapping screws and the supplied drill bit provided by the MED-EL surgical toolkit were not used in the middle fossa approach described here. Instead, the unique use of self-drilling screws was much faster by removing the need for a torque wrench or a compatible drill. In our series with up to 71 months follow-up, no issues with screws loosening, or a decrease in audiometric gain were seen. The primary disadvantage of the self-drilling screws was that the heads could break under excessive torque. Therefore, the technique was modified to use BCI lifts and a gentle tightening technique as described. Before the availability of formal BCI lifts by MED-EL, single rings cut from Synthes MatrixMIDFACE plates were used to substitute BCI lifts.

In general, complications following BB-BCI are infrequent. A systematic review in 2016 reviewed 117 patients following BB-BCI implantation and found six minor adverse events and a revision surgery rate of 0.85% [9]. A prospective European study with 1 year follow-up reported no revision surgery, device failure, or skin injury [25]. Furthermore, no complications were encountered in our institutional review.

The low complication rate associated with the BB-BCI is complemented by the advantages of early activation and positive audiometric outcomes. BB-BCI does not require osseointegration as only vibrational energy is transmitted through the anchor screws [9, 17]. Therefore, the patients with BB-BCI undergo activation much earlier than those with percutaneous BCIs. While some authors activate BB-BCI at postoperative day seven,^14^ it is standard practice at our centre to activate at 2 weeks post-implantation.

Regarding audiometric outcomes, Schmerber et al. evaluated 28 adults following BB-BCI implantation at 1-year post-operatively. Patients showed improvements in audiometric thresholds and speech intelligibility for speech in quiet and noise, and good patient satisfaction was reported [25]. In children implanted with the BB-BCI, one study followed 12 patients aged 5–17 years prospectively with a follow-up of 3 months and showed excellent patient satisfaction and a high average of hours of device use per day [26]. In a systematic review, Sprinzl et al. identified seven studies (with a total of 58 patients) that reported audiometric outcomes of adult patients with conductive or mixed hearing loss [9]. The functional gain was found to range from 24 to 37 dB. Similarly, Zernotti et al. identified five studies (with a total of 20 patients) in a systematic review, citing a functional gain ranging from 24 to 43 dB [11]. Therefore, the functional gain of 39.6 dB reported in the present study is favourable and comparable to what has been reported in the literature using the BAHA and the BB-BCI implanted via transmastoid or retrosigmoid approaches. Future work will include detailed analyses of the audiometric data and sound quality measures, which will be published in separate reports.

## Conclusion

The middle fossa technique with self-drilling screws is a safe alternative approach to BONEBRIDGE implantation. An annotated video highlighting the surgical technique has been included as a supplement to the study. No audiometric or surgical complications were noted after an average follow-up of 29 months. The novel preoperative modelling precluded the need for image guidance or templates. Although the approach is efficient with excellent cosmesis, surgeons opting to use this approach should be familiar with perioperative planning and comfortable with dura, venous sinuses, and middle fossa anatomy.

## Additional file

Additional file 1: Annotated video summarizing the surgical steps for the middle fossa approach with self-drilling screws for BONEBRIDGE implantation. (mp4 517 kb)

## Data Availability

The dataset generated during the current study is available from the corresponding author on reasonable request.

## Abbreviations

3D: Three -dimensional
BAHA: Bone anchored hearing aid
BB-BCI: BONEBRIDGE bone conduction implant
BC-FMT: Bone Conduction-Floating Mass Transducer
BCI: Bone conduction implant
CT: Computed tomography
dB HL: Decibels hearing level
MRI: Magnetic resonance imaging
OR: Operating room
ROI: Region of interest
SSD: Single sided deafness
US: United States
